# Design and assembly of a chemically switchable and fluorescently traceable light-driven proton pump system for bionanotechnological applications

**DOI:** 10.1038/s41598-018-37260-9

**Published:** 2019-01-31

**Authors:** S. Hirschi, N. Fischer, D. Kalbermatter, P. R. Laskowski, Z. Ucurum, D. J. Müller, D. Fotiadis

**Affiliations:** 1 Institute of Biochemistry and Molecular Medicine, University of Bern, Bern, Switzerland; 2 Department of Biosystems Science and Engineering, ETH Zürich, Basel, Switzerland

## Abstract

Energy-supplying modules are essential building blocks for the assembly of functional multicomponent nanoreactors in synthetic biology. Proteorhodopsin, a light-driven proton pump, is an ideal candidate to provide the required energy in form of an electrochemical proton gradient. Here we present an advanced proteoliposome system equipped with a chemically on-off switchable proteorhodopsin variant. The proton pump was engineered to optimize the specificity and efficiency of chemical deactivation and reactivation. To optically track and characterize the proteoliposome system using fluorescence microscopy and nanoparticle tracking analysis, fluorescenlty labelled lipids were implemented. Fluorescence is a highly valuable feature that enables detection and tracking of nanoreactors in complex media. Cryo-transmission electron microscopy, and correlative atomic force and confocal microscopy revealed that our procedure yields polylamellar proteoliposomes, which exhibit enhanced mechanical stability. The combination of these features makes the presented energizing system a promising foundation for the engineering of complex nanoreactors.

## Introduction

Designing, creating and manipulating existing or novel artificial biological systems is the core concept of synthetic biology^1^. Natural biological systems can be equipped with new desired features using genetic modifications or by introducing foreign natural or artificial elements. The engineering of biomimetic or completely new-to-nature systems based on living organisms is termed ‘top-down’ approach^1^. ‘Bottom-up’ on the other hand refers to the assembly of isolated and well-characterized biomolecules into a functional molecular system^1^. The use of engineered products range from direct uses in biotechnology and medicine to applications as valuable tools in biological research^2–4^. Simple bottom-up assembled biomolecular nanoreactors generally consist of a container in the nanoscale (e.g., vesicular structures such as liposomes or polymersomes) with functional protein modules embedded in the membrane and/or encapsulated within the reactor^5^. The functionality and in turn the scope of application of such systems is determined by the assembled modules. Proteins are undoubtedly the biomolecules with the most diverse functions. Furthermore, most proteins can easily be produced in high quantities and engineered to meet specific requirements, thus making them ideal modular entities. Basic applications of the described nanoreactors are the self-sustained uptake of solutes with high specificity and efficiency, or the triggered release of encapsulated or *in situ* produced compounds^5,6^. Energizing or energy converting modules in the membrane are essential for supplying the required energy for subsequent transport and metabolizing modules. One of the most prominent examples represents the co-reconstitution of the light-driven proton pump bacteriorhodopsin and the mitochondrial ATP synthase into lipid vesicles to drive ATP synthesis by a light-induced proton gradient^7^. A more complete overview and specific examples of applications involving energizing and transport modules can be found in a recent review^5^. In nature, energy is commonly stored in form of electrochemical gradients across cell membranes that can be used to drive the transport of a vast number of substrates into or out of the cell^8^. Based on this concept, light-driven proton pumps are efficient energy converting modules to provide systems with energy in form of proton gradients. The membrane protein proteorhodopsin (PR) is a well-characterized light-driven proton pump^9^ that can easily be genetically engineered and overexpressed in *Escherichia coli*^10,11^. These features make PR an ideal energizing module candidate for biomolecular nanoreactors.

Membrane protein orientations in proteoliposomes reconstituted from fully solubilized material is commonly random (i.e., cytoplasmic side of the protein facing inward or outward) but can have slight preferences^12–14^. Opposing orientations of light-driven proton pumps in membranes lead to functional short-circuits that prevent the establishment of electrochemical proton gradients. A possible solution to resolving such short-circuits is the selective deactivation (switching off) of one population of proteins by chemical modification. The latter is a versatile tool to investigate the working mechanisms of membrane proteins and to control their activities^15,16^. Alternatively, specific membrane proteins can be controlled by light-activated ligands that act as photoswitches and which are also of particular interest in the context of photopharmacology^17^. In recent work we demonstrated the extended functionality of the PR-N221C mutant (N220C in the reference) that can be repeatedly switched off and on by chemical modification with the sulfhydryl-modifying agent sodium (2-sulfonatoethyl) methanethiosulfonate (MTSES) and reduction with β-mercaptoethanol (β-ME)^10^. This is a valuable approach to prevent unintended PR activation, e.g., during imaging of PR containing samples with a light or confocal laser scanning microscope, or to control its activity in situations without possibility to regulate the source of illumination, e.g. when using sunlight. Importantly, chemical protein modification provides a solution to selectively inactivate undesirably oriented PR molecules in proteoliposomes, thus preventing a functional short-circuit^5^.

Fluorescence is a highly valuable feature for detection and tracking of objects in the micro- or nanoscale. Due to the high detection sensitivity of commonly used instruments, such as fluorescence microscopy, traces of fluorophores are usually sufficient. Nanoparticle tracking analysis (NTA) is a powerful tool for the analysis of polydisperse nanoparticles as small as 50 nm^18^. It detects nanoparticles based on scattered light and traces them over a certain distance to calculate their size. This can be combined with fluorescently labelled nanoparticles to specifically detect, track and analyse a subpopulation of particles. The use of NTA and fluorescence microscopy opens the possibility to detect and track a population of fluorescently labelled nanoreactors in complex environmental or biological samples.

Here we optimized the specificity and efficiency of deactivation and regeneration of the PR-N221C mutant as a module for the assembly of future biomolecular nanoreactors. Reaction conditions were improved to achieve an essentially complete recovery of the proton pumping activity after regeneration. The homogeneity of the protein sample was improved by truncation of the N-terminal signal sequence (PRΔ18) while maintaining full functionality. Importantly, optical traceability of the proteoliposomes using fluorescence detection instruments was made possible by the supplementation of fluorescently labelled lipids in the assembly process. Particles analysed with NTA could be specifically tracked and were homogenous in regard to size distribution. The presented reconstitution procedure yielded polylamellar proteoliposomes as shown by cryo-transmission electron microscopy (cryo-TEM). The three-dimensional morphology of proteoliposomes adsorbed on a solid support was assessed using atomic force microscopy (AFM) and correlated with fluorescence microscopy for tracking. Adsorption and successful imaging by AFM of intact particles indicated mechanical stability. Combined, these properties established the presented proteoliposomes as a solid foundation for complex synthetic systems.

## Methods

### Cloning

The wild-type gene of green-light absorbing PR (GenBank: AY601905.1) without signal sequence (PR∆18) was cloned into the pZUDF21 vector^19^. Heterologous overexpression in *E. coli* yielded a recombinant protein containing a C-terminal decahistidine-tag suitable for metal affinity chromatography purification. The QuikChange Lightning Multi Site-Directed Mutagenesis Kit (Agilent) was used to generate the PRΔ18 mutants PR∆18-C176S, PR∆18-N221C and PR∆18-C176S-N221C.

### Overexpression of PR in *E. coli*

PR constructs were overexpressed as described previously^10^. Briefly, *E. coli* BL21(DE3) Rosetta2 cells were transformed with the pZUDF21 construct containing the PR gene variants. After initial colony selection, the best expressing clone was used for overexpression. Cells were grown at 37 °C and 180 rpm (Multitron, Infors HT) to an OD_600_ of 0.5 and further at 20 °C to an OD_600_ of 0.75. Expression was then induced with 0.1 mM isopropyl-β-D-thiogalactopyranoside (IPTG) and 5 µM all-*trans* retinal (dissolved in ethanol), followed by further incubation at 20 °C and 180 rpm overnight. Cells were harvested by centrifugation at 4 °C and 10,000 × g for 5 min (Sorvall RC-5B centrifuge, DuPont Instruments), resuspended in 50 mM Tris-HCl pH 8, 450 mM NaCl and stored at −20 °C until further use.

### Purification of PR

Cells were thawed and then lysed by five passes through a Microfluidizer (M-110P Microfluidizer, Microfluidics) at 1,500 bar. Unlysed cells were removed by centrifugation (Sorvall RC-5B centrifuge, DuPont Instruments) at 4 °C and 10,000 × g for 10 min. The supernatant containing the cell membranes was pelleted by ultracentrifugation (Optima L-90K ultracentrifuge, Beckman Coulter) at 4 °C and 150,000 × g for 1 h. Pellets were homogenized and washed twice with 50 mM Tris-HCl pH 8, 450 mM NaCl by repeating the previous centrifugation. Finally, pellets were homogenized in solubilization buffer (20 mM 4-(2-hydroxyethyl)-1-piperazine ethanesulfonic acid (HEPES)-NaOH pH 7.5, 300 mM NaCl, 10% (v/v) glycerol, 0.5 mM tris (2-carboxyethyl) phosphine hydrochloride (TCEP)) and stored at −80 °C in aliquots corresponding to one liter of bacterial culture each. Membrane aliquots were solubilized in a total of 7 mL solubilization buffer containing 3% (w/v) *n*-octyl-β-D-glucopyranoside (OG) at 4 °C on a turnover shaker overnight. The solubilized membranes were ultracentrifuged (Optima L-90K ultracentrifuge, Beckman Coulter) at 25 °C and 100,000 × g for 45 min and the supernatant was diluted with 7 mL wash buffer (i.e., solubilization buffer containing 60 mM imidazole). The protein was bound to 0.5 mL equilibrated nickel-nitrilotriacetic acid (Ni-NTA) resin (PerfectPro Ni-NTA System, 5Prime) by repeated loading on a gravity flow column for 3 h at room temperature (RT). The column was washed with 20 mL wash buffer including 1% (w/v) OG. The protein was eluted with solubilization buffer containing 400 mM imidazole and 1% (w/v) OG. Colorless fractions were discarded and red colored fractions pooled. The concentrations of PR containing fractions were determined spectrophotometrically (Nanodrop 1000, Thermo Scientific) at the absorption maximum of the retinal Schiff base 520 nm (extinction coefficient 45,000 M^−1^ cm^−1^).

### Detergent-mediated reconstitution of PR into preformed liposomes

10 mg 1,2-dioleoyl-*sn*-glycero-3-phosphocholine (DOPC, Avanti Polar Lipids) dissolved in chloroform were dried under a stream of nitrogen in a 10 mL round bottom flask. For fluorescently labelled proteoliposomes 0.028 mg or 0.112 mg (0.25 mol% or 1 mol%) 1,2-dipalmitoyl-*sn*-glycero-3-phosphoethanolamine-*N*-(7-nitro-2-1,3-benzoxadiazol-4-yl) ammonium salt (NBD-PE, Avanti Polar Lipids) dissolved in chloroform were added before drying. Remaining chloroform traces were removed under vacuum overnight in a desiccator. The lipid was hydrated with 2 mL potassium phosphate buffer (20 mM KP_i_ pH 7.2, 100 mM KCl, 1 mM TCEP) on a thermomixer (Thermomixer, Eppendorf) at RT and 800 rpm for 15 min. The liposomes were destabilized by adding OG to 0.75% (w/v) and further shaking at RT and 800 rpm for 15 min. 400 µg purified PR were added (final OG concentration 0.8% (w/v)) to the destabilized liposomes. The suspension was extruded (Mini-Extruder, Avanti Polar Lipids) by 19 passages through a 200 nm pore size polycarbonate membrane. The suspension was dialyzed in 14 kDa molecular weight cut-off dialysis tubing (Membra-cel, Carl Roth) against 2 L of 20 mM Tris-HCl pH 7.5 with magnetic stirring overnight at RT.

### Photoactivity measurements

**Figure 1:**
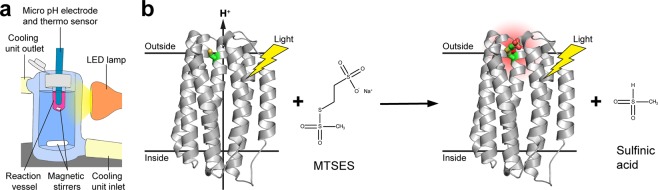
Experimental setup for photoactivity measurements and schematic reaction mechanism of MTSES. (**a**) Experimental setup for photoactivity measurements with PR proteoliposomes. The sample is water-cooled and stirred with a magnetic stirrer in a transparent reaction vessel during measurements. Illumination induced pH changes in the extravesicular solution are recorded using a micro pH electrode with integrated temperature sensor. Illumination is provided by a LED lamp. (**b**) Schematic illustration of the MTSES reaction mechanism with an engineered cysteine residue in PR (shown as sticks; carbon, oxygen and sulfur atoms are colored in green, red and yellow, respectively). Lipid bilayer of proteoliposomes is represented by horizontal black lines and the relative orientation is indicated (i.e., outside or inside the vesicle). Boundaries are based on the OPM (orientation of proteins in membranes) database calculations (http://opm.phar.umich.edu).

Dialyzed proteoliposomes were harvested by ultracentrifugation at 4 °C and 200,000 × g (Optima MAX-XP, Beckman Coulter) for 20 min, washed twice with measuring solution (150 mM NaCl pH 7.4) and resuspended in 800 μL. To assess the light-driven proton pumping activity of PR, the pH value of the extravesicular medium was measured in a transparent 2 mL tube using a micro pH-electrode with integrated temperature sensor (InLab Micro Pro, Mettler Toledo). The sample was stirred and maintained at 18 °C in a cooling water bath (Julabo F10, Gemini) with a cooling vessel (see Fig. 1a for a schematic representation of the setup). pH data were recorded automatically in 30 s intervals using the LabX direct pH 2.3 software (Mettler Toledo) on a computer connected to the pH-meter (SevenCompact pH-meter, Mettler Toledo). The sample was illuminated with a 2 W, 3000 K, warm white LED lamp (JANSÖ, IKEA) and protected from exterior light. The pH changes were recorded for four light-dark cycles with 15 min of illumination and darkness each after 15 min of initial dark adaptation.

Photoactivity measurements of the same sample were recorded before, after 8 mM MTSES and after 75 mM β-ME treatment. For treatment, proteoliposomes were centrifuged (Optima MAX-XP, Beckman Coulter) and resuspended in 800 µL modification buffer (50 mM 3-(*N*-morpholino) propanesulfonic acid (MOPS)-NaOH pH 7, 150 mM NaCl) containing 8 mM MTSES in water or dimethyl sulfoxide (DMSO, final DMSO concentration 0.5% (v/v)). The reaction was incubated shaking at 25 °C on a thermomixer at 900 rpm for 45 min (the general mechanism of the reaction is shown in Fig. 1b). Proteoliposomes were washed twice in measuring solution, resuspended in 800 µL of the same solution and the photoactivity was measured again. MTSES was removed from the cysteine residue by treatment with 75 mM β-ME under identical conditions as used for MTSES modification. After the reaction, proteoliposomes were washed twice with measuring solution, resuspended in 800 μL of the same solution and the photoactivity was measured a last time.

### Data correction

Raw data of photoactivity measurements were corrected as described previously^10,11^. Briefly, the first of four consecutively recorded peaks was discarded due to strong initial drift and data were corrected by subtracting a background based on measurements without proteoliposomes. Continuous drift of the raw data was corrected by subtracting a continuous piecewise linear function, defined by two sequential starting points of an illumination cycle.

### UV-Vis spectroscopy

UV-Vis spectra of purified PR and of PR proteoliposomes before, after MTSES and after β-ME treatment were measured using a UV-1600PC spectrophotometer (VWR) in conjunction with the M.Wave Professional software (version 1.0.20). Spectra were recorded from 200 nm to 800 nm with 1 nm intervals. All measurements were performed at pH 7.5.

### Nanoparticle tracking analysis

Size distribution, concentration and fluorescence of PRΔ18-C176S-N221C proteoliposomes labelled with 0.25 mol% or 1 mol% NBD-PE were assessed by nanoparticle tracking analysis (NTA). A NanoSight NS300 instrument (Malvern Panalytical) equipped with a flow-cell top plate and a 488 nm laser module at 25 °C was used. In light scatter mode the total number of particles was measured without the use of an optical filter. For fluorescence measurements the 488 nm laser module was used in conjunction with a 500 nm low-pass filter. Samples were applied with a constant flow rate using the supplied syringe-pump (pump speed 100). Proteoliposomes were diluted 1:10,000 in 150 mM NaCl (filtered with a 0.2 μm filter) prior to NTA measurements. Three to five videos of 60 s were recorded per sample with a sCMOS camera at 25 frames per second (1,498 frames per video) and data were analyzed with the NTA software 3.1 (Build 3.1.54).

### Cryo-TEM

PRΔ18-C176S-N221C NBD-PE proteoliposomes (2.5 μl) were applied to a previously glow-discharged (10 mA for 120 s) holey carbon grid (Cu R2/1 200 mesh, Quantifoil). The grid was blotted on both sides for 4 s in a Vitrobot (FEI) at 100% humidity and 4 °C, then plunged into cooled liquid ethane. Cryo-TEM images were collected at liquid nitrogen temperature on a Tecnai F20 electron microscope (FEI) operated at 200 kV equipped with a FEI Falcon 3 direct electron detector at a magnification of 50,000× (corresponding to a step size of 2.08 Å/pixel at the specimen level). The defocus value of the displayed electron micrograph was −2.0 μm and the exposure time was 2 s resulting in a total electron dose of ~24 e^−^/Å^2^.

### Correlative atomic force and fluorescence microscopy

Proteoliposomes were diluted with imaging buffer (20 mM HEPES-NaOH, pH 7.4, 30 mM KCl) 500 times for morphology analysis and 10,000 times for AFM-fluorescence microscopy correlation, and adsorbed on freshly cleaved mica for 20 min. The sample was then imaged with an atomic force microscope (BioScope Resolve, Bruker) installed on the stage of an inverted confocal microscope (LSM 800, Carl Zeiss). During confocal imaging a 10 mW, 488 nm laser at 5% power and a 1 airy unit pinhole was used. Images were acquired with a 63x water immersion lens (421787-9970-799 objective, NA 1.20, Carl Zeiss). The area scanned with the confocal microscope was subsequently imaged by AFM. The atomic force microscope was equipped with a 100 × 100 × 15 µm (x, y, z) piezoelectric scanner and was operated with NanoScope 9.4R3 software (Bruker) in ‘Peak Force QNM’ imaging mode. All images were acquired with rectangular cantilevers (AC40, Olympus) with a nominal spring constant of 0.09 N m^–1^ and resonance frequency in water of 25 kHz. AFM cantilevers were calibrated by ramping on a solid surface followed by thermal tuning. Imaging was performed by applying 100 pN imaging force, 2 kHz oscillation frequency, 0.7–1 Hz scanning rate and 200–300 nm amplitude. Feedback gain was optimized to achieve low contrast of the error signal. All imaging was performed at room temperature.

## Results

### Optimization of PR as controllable energizing module

**Figure 2:**
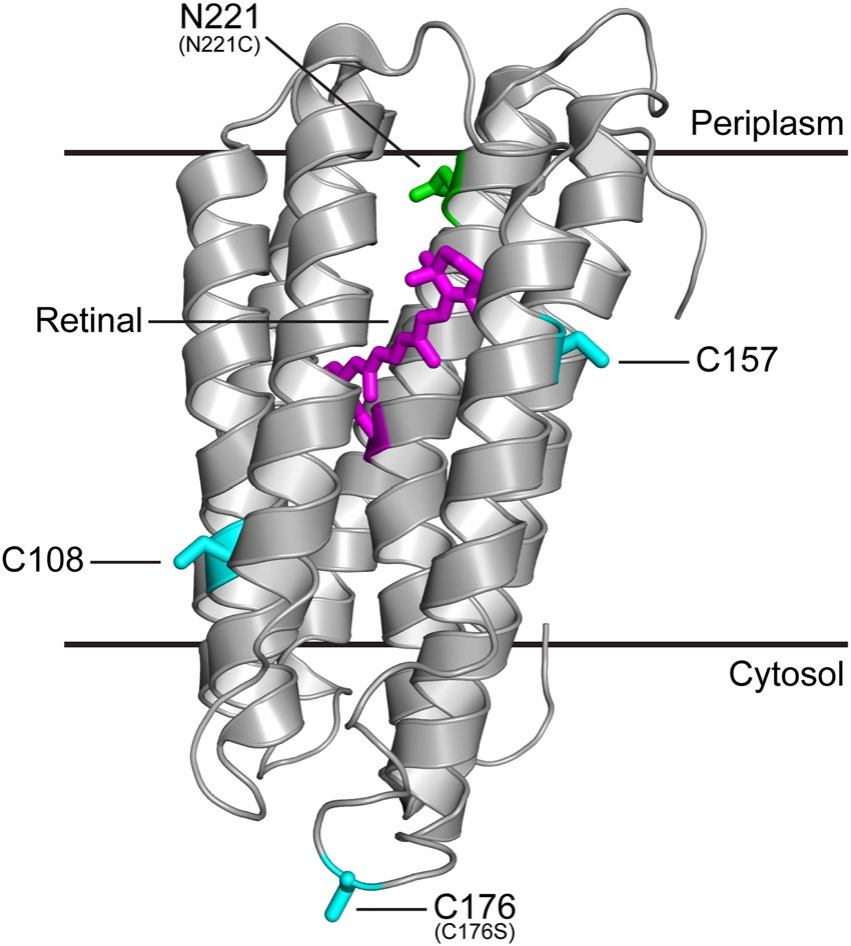
PR structure with endogenous cysteine residues and other important features. Ribbon representation of the PR solution NMR structure (PDB: 2L6X^20^) showing the retinal cofactor (in magenta), the three endogenous cysteines (in cyan), including C176 that was mutated to a serine, and asparagine N221 that was mutated to a cysteine (in green). Lipid bilayer boundaries are indicated and based on the OPM (orientation of proteins in membranes) database calculations (http://opm.phar.umich.edu).

We recently demonstrated the possibility of chemically switching off and on the proton pumping activity of the engineered PR-N221C mutant based on the chemical modification of the site-specifically introduced cysteine^10^. Wild-type PR contains three endogenous cysteines that could potentially interfere with the chemical modification of N221C (Fig. 2) depending on the experimental set-up. In order to ensure the specificity of the thiol modification with residue N221C only, the solvent accessibility of all cysteines in PR was assessed. The structure of proteorhodopsin (PDB: 2L6X^20^) is depicted in Fig. 2 with all cysteines (in cyan) and asparagine N221 (in green) emphasized. Only one of the three naturally occurring cysteines, i.e., C176, was considered accessible enough to potentially interfere with the chemical modification of N221C and was therefore conservatively mutated to a serine. The remaining two cysteines are exposed on the hydrophobic part of the protein protruding into the lipid bilayer and should thus be inaccessible for hydrophilic modifying reagents such as MTSES.

**Figure 3:**
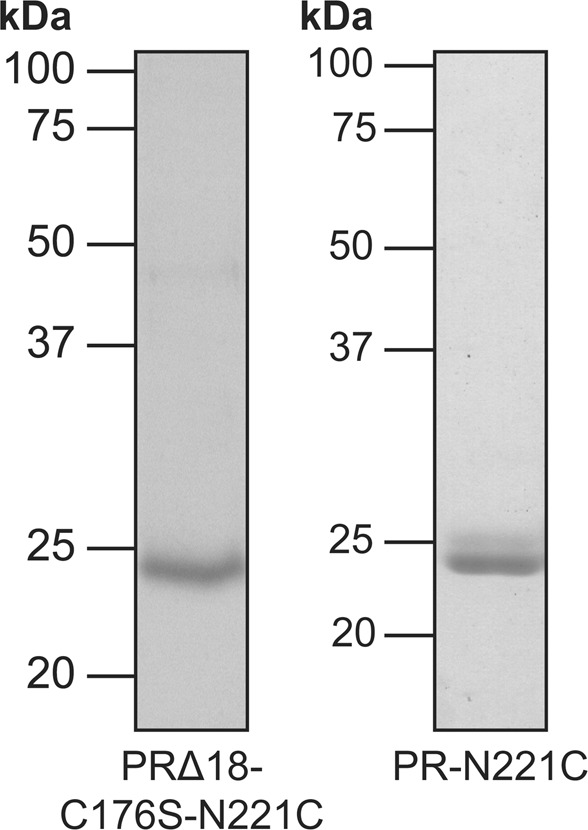
SDS-PAGE of PRΔ18-C176S-N221C and PR-N221C. Purified PRΔ18-C176S-N221C (left) runs as a single band slightly below 25 kDa as compared to PR-N221C (right), which runs as a double band around 25 kDa.

**Figure 4:**
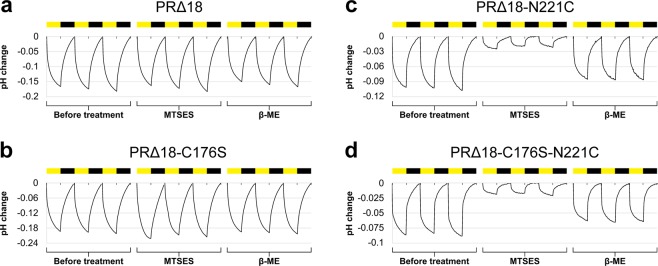
Photoactivity of proteoliposomes containing PRΔ18 versions. Background and drift corrected photoactivity measurement of PR∆18 (**a**), PR∆18-C176S (**b**), PR∆18-N221C (**c**) and PR∆18-C176S-N221C (**d**) proteoliposomes. Each panel shows consecutive measurements of the same sample: untreated, 8 mM MTSES and 75 mM β-ME treated. The yellow and black bars indicate 15 min light and dark cycles. A representative experiment for each PR mutant is shown.

**Figure 5:**
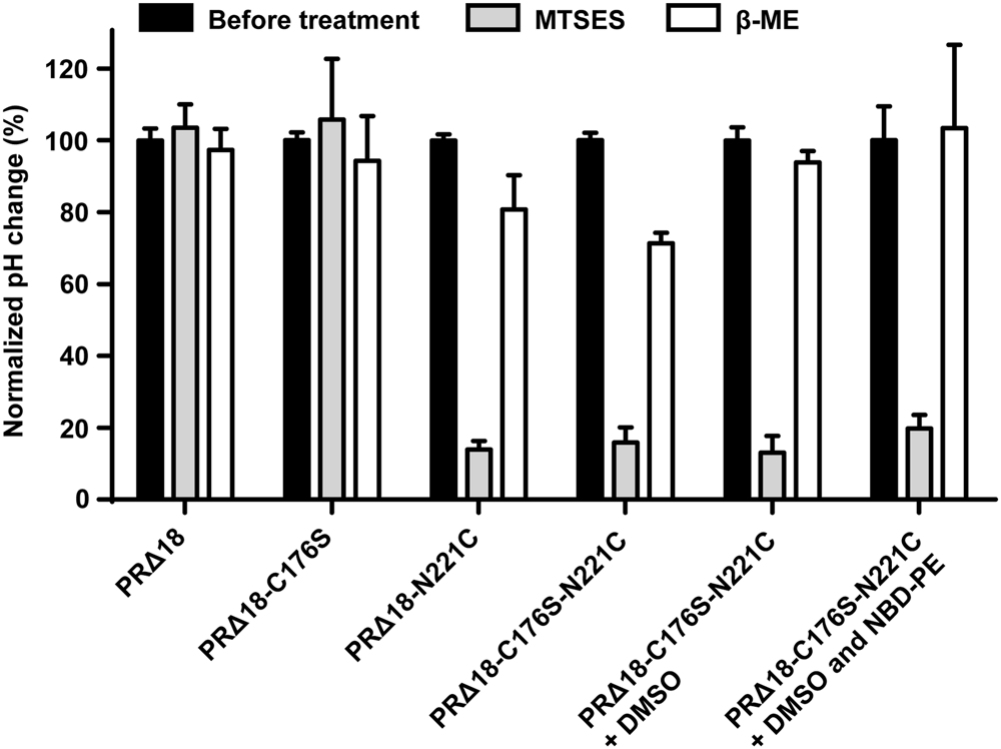
Photoactivity measurements of PR proteoliposomes. Photoactivity of PR∆18 and PR∆18-mutants proteoliposomes was measured before, after 8 mM MTSES and after 75 mM β-ME treatment. pH changes of the samples are normalized to the measurements before treatment. Each bar represents at least three independent experiments in triplicates: PR∆18-C176S five independent experiments; PR∆18, PR∆18-N221C and PR∆18-C176S-N221C three independent experiments; PR∆18-C176S-N221C + DMSO (8 mM MTSES and 0.5% (v/v) DMSO treated) four independent experiments, and PR∆18-C176S-N221C + DMSO and NBD-PE (proteoliposomes including 0.25 mol% NBD-PE lipid, treated with 8 mM MTSES and 0.5% (v/v) DMSO) four independent experiments. Error bars indicate standard deviations.

Truncated and mutated PR versions were expressed, purified and reconstituted into preformed liposomes and assessed for their photoactivity. Average maximal pH changes of all PR versions are summarized in Table 1. Truncation and introduction of the C176S mutation did not negatively affect the proton pumping activity of PR. The final construct PRΔ18-C176S-N221C was reconstituted with virtually the same efficiency as compared to the full length wild-type construct (Table S1). The maximal absorbance of purified PRΔ18-C176S-N221C (Fig. S1) is 520 nm and when reconstituted into DOPC liposomes blue-shifted to 510 nm. However, MTSES and β-ME treatment seem to have no significant effect on the maximal absorption wavelength of reconstituted protein yielding 508 nm and 509 nm, respectively. Notably, removal of the N-terminal signal sequence yields a homogeneous PRΔ18-C176S-N221C sample, running as a single band on SDS-PAGE compared to PR-N221C, which runs as a double band because of incomplete removal of the signal sequence in *E. coli* (Fig. 3). Background and drift corrected photoactivity measurements of PRΔ18, PRΔ18-C176S, PRΔ18-N221C and PRΔ18-C176S-N221C proteoliposomes before, after 8 mM MTSES and after 75 mM β-ME treatment are shown in Fig. 4 as complete pH traces. A representative experiment of the same sample undergoing sequential MTSES and β-ME treatments is displayed for each PR mutant. The activities of PR∆18 and PR∆18-C176S were not affected by MTSES or β-ME (Fig. 4a,b). Both PR variants containing the N221C mutation could be inactivated by chemical modification with MTSES and regenerated with β-ME with comparable efficiencies (Fig. 4c,d). Data of all photoactivity measurements are summarized in Fig. 5 where pH changes were normalized to the untreated samples. The proton pumping activities of PR∆18-N221C and PR∆18-C176S-N221C could be reduced to less than 20% after MTSES treatment and regenerated to around 80% by reduction with β-ME. Importantly, almost complete regeneration of the initial activity was achieved when a final concentration of 0.5% (v/v) DMSO was included in the MTSES reaction. PR proteoliposomes labelled with 0.25 mol% of the fluorescent lipid NBD-PE exhibit a very similar photoactivity pattern. The additional NBD-PE does not adversely affect the MTSES reaction and the beneficial effect of DMSO on the regeneration of the photoactivity is also preserved.

**Table 1:** Photoactivity measurements of PR proteoliposomes.

PR version	n	∆pH	SD
PR-wt*	6	−0.11	±0.02
PR-N221C*	3	−0.07	±0.02
PR∆18	3	−0.15	±0.03
PR∆18-C176S	5	−0.17	±0.03
PR∆18-N221C	3	−0.09	±0.01
PR∆18-C176S-N221C	16	−0.09	±0.02

*From reference^10^.

n: number of independent experiments, each experiment in triplicate.

ΔpH: average of maximal pH changes.

SD: standard deviation.

### Traceability of fluorescently labelled proteoliposomes

**Figure 6:**
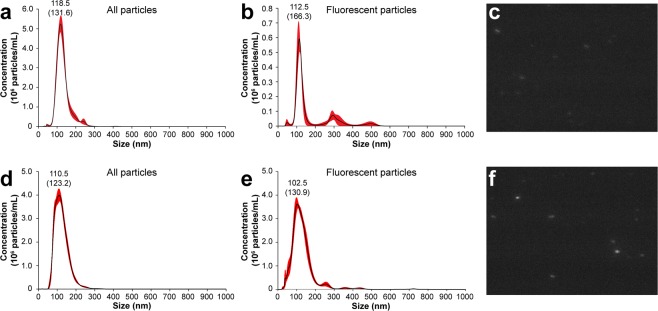
Nanoparticle tracking analysis (NTA) of NBD-PE labelled PR∆18-C176S-N221C proteoliposomes. Proteoliposomes containing 0.25 mol% NBD-PE (**a**–**c**) and 1 mol% NBD-PE (**d**–**f**) were analysed using a 488 nm laser module in light scatter mode (**a** and **d**) and in fluorescence mode with a 500 nm low-pass band filter (**b** and **e**). Distribution of particle concentrations shown represent 1:10,000 diluted samples. Standard deviations are indicated in red and the most frequent particle size (mode) and the mean (in brackets) with a label. Measurements in light scatter mode (**a** and **d**) reveal homogeneous proteoliposome populations with a mean particle size of 131.6 ± 2.0 nm and mode of 118.5 nm for the 0.25 mol% NBD-PE sample, and mean particle size of 123.2 ± 0.5 nm and mode of 110.5 nm for the 1 mol% NBD-PE sample. Fluorescent particles measured in fluorescence mode (**b** and **e**) have a similar distribution with a mean particle size of 166.3 ± 14.9 nm and mode of 112.5 nm for the 0.25 mol% NBD-PE sample and mean of 130.9 ± 3.6 nm and mode of 102.5 nm for the 1 mol% NBD-PE sample. Snapshot of a NTA video (**c** and **f**) depicting fluorescent PR proteoliposomes of the corresponding samples in the scattering volume of the laser (cylinder of approximately 70 μm diameter and 80 μm length).

Traceability of PR∆18-C176S-N221C proteoliposomes supplemented with 0.25 mol% NBD-PE was assessed using nanoparticle tracking analysis (NTA) (Fig. 6a–c). First the size distribution and total concentration of proteoliposomes was recorded in light scatter mode (Fig. 6a). The measurements revealed a homogeneous sample with a mean particle size of 131.6 ± 2.0 nm and mode (most frequent population of particle sizes) of 118.5 nm. Fluorescent particles (Fig. 6b) exhibited a similar distribution with a mean particle size of 166.3 ± 14.9 nm and mode of 112.5 nm. The total proteoliposome concentration is represented by the area under the curve of the light scattering experiment and equals 3.20 × 10^12^ ± 3.82 × 10^10^ particles/mL. When compared to the concentration of fluorescent particles, 2.85 × 10^11^ ± 7.62 × 10^9^ particles/mL, it is notable that apparently only about 10% of all particles are labelled. Eventhough faint fluorescent particles are visible by eye (Fig. 6c), they are mostly not detected by the analysis software, resulting in an underestimated particle concentration. To compensate for the weak fluorescent signal, proteoliposomes were supplemented with 1 mol% NBD-PE instead (Fig. 6d–f). A very similar particle size distribution was observed compared to the lower NBD-PE concentration. Mean particle size was 123.2 ± 0.5 nm with a mode of 110.5 nm in light scatter mode (Fig. 6d), and mean particle size of 130.9 ± 3.6 nm and a mode of 102.5 nm in fluorescence mode (Fig. 6e). Concentration of total and of fluorescent particles were almost identical with 3.27 × 10^12^ ± 2.29 × 10^10^ particles/mL and 3.37 × 10^12^ ± 2.38 × 10^10^ particles/mL, respectively. Importantly, this shows full detection of all particles by NTA when using a higher concentration of NBD-PE.

### Mechanical stability of polylamellar proteoliposomes

**Figure 7:**
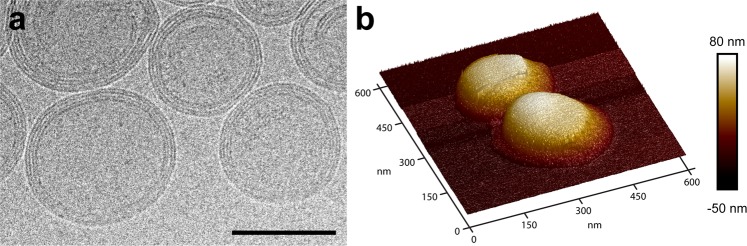
Structural analysis of PR∆18-C176S-N221C NBD-PE proteoliposomes by cryo-TEM and AFM. (**a**) Cryo-TEM image of PR NBD-PE proteoliposomes revealing polylamellar vesicles. Scale bar is 100 nm. (**b**) Three dimensional representation of an AFM topograph depicting two intact proteoliposomes adsorbed to mica. The topograph was recorded in buffer solution at room temperature.

**Figure 8:**
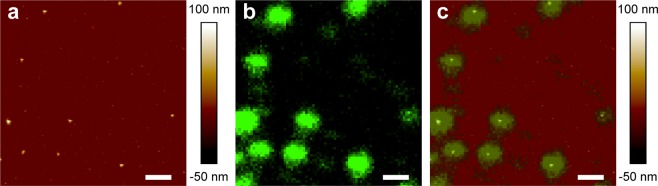
PR∆18-C176S-N221C NBD-PE proteoliposomes visualised with correlative AFM and confocal fluorescence microscopy. AFM topograph of PR NBD-PE proteoliposomes adsorbed to mica (**a**) and the same area scanned by fluorescence microscopy (**b**). Merged fluorescence and AFM topograph (**c**) correlating the fluorescence signal with AFM imaged vesicles. Scale bars represent 1 μm.

Cryo-TEM was used to gain insight into the internal structure of PR∆18-C176S-N221C NBD-PE proteoliposomes and to evaluate if the vesicles are uni- or polylamellar. Micrographs show that the vast majority of proteoliposomes exhibit polylamellarity with multiple densely packed lipid bilayers clearly visible inside the vesicles (Fig. 7a). The refractive intensity from NTA measurements (Fig. S2) indicates a homogeneous distribution in regard to the density (equivalent to lamellarity) of the tracked particles. Polylamellar liposomes are assumed to be more mechanically and osmotically stable than unilamellar liposomes^21,22^. It was thus evaluated if these vesicles can be adsorbed onto a flat surface and imaged by AFM without rupturing, a feature that unilamelar proteoliposomes do not exhibit^23^. The morphology of polylamellar vesicles adsorbed onto mica was assessed using AFM (Fig. 7b), which revealed mostly intact, slightly flattened vesicles with heights of up to 90 nm. With recently introduced combinations of AFM and confocal microscopy^24^ it is possible to correlate AFM topographs with fluorescence microscopy images from the same area. Fluorescently labelled (0.25 mol% NBD-PE), polylamellar PR proteoliposomes adsorbed onto mica were imaged by AFM (Fig. 8a) and confocal microscopy (Fig. 8b). The merged image (Fig. 8c) nicely displays the local correlation of fluorescence and intact proteoliposomes. Compared to NTA measurements, which uses a less sensitive detection system, the majority of vesicles appear to be fluorescent, indicating efficient labelling.

## Discussion

Inhomogeneities of the purified PR sample due to incomplete cleavage of the N-terminal signal sequence during overexpression in *E. coli* could be eliminated using the truncated PRΔ18 version (Fig. 3). While yielding a nicely homogeneous protein sample, truncated PRΔ18 versions all exhibited light-dependent proton pumping activity (Table 1). PR proteoliposomes temporarily acidified the extravesicular solution upon illumination (Table 1 and Fig. 4). This indicates the same orientation of PR in liposomes as previously observed, i.e., right-side-out^10^, regardless of the presence or absence of the N-terminal signal sequence. Furthermore, inhibition of the proton pumping activity with MTSES by over 80% suggests an efficient unilateral reconstitution of PR in the proteoliposome membrane. Introduction of the C176S mutation did not negatively affect the function of PR, nor the efficiency of chemical inactivation or regeneration (Figs 4 and 5). It is essential for the prevention of functional short-circuits that the deactivation of unfavourably oriented PR populations is not hindered by any solvent accessible cysteines besides N221C. Randomized orientations of membrane proteins in proteoliposomes are commonly observed as a result of reconstitutions starting with fully solubilized protein-lipid-detergent mixtures^12,13^. Substitution of cysteine C176 with a serine excludes the possibility of unspecific chemical modification in cases where the residue might be exposed on the outside of proteoliposomes. We found that the efficiency with which the cysteine modification can be reversed is significantly increased in the presence of 0.5% (v/v) DMSO during the reaction with MTSES. Importantly, it was possible to recover the initial activity of the PR proteoliposomes almost completely using these newly established conditions. This observation could potentially prove useful for other reversible labelling reactions involving membrane proteins in membrane environments.

Using nanoparticle tracking analysis (NTA) we investigated the possibility of optically tracking fluorescently labelled proteoliposomes in aqueous systems. PR proteoliposomes supplemented with 0.25 mol% NBD-PE were analyzed by NTA in light scatter and fluorescence mode (Fig. 6a,b). Measurements showed a homogeneous particle population that exhibits a fluorescent signal. Faint fluorescent particles can even be observed by eye (Fig. 6c). To improve the detection of fluorescent particles, e.g. for quantitative analysis, the concentration of NBD-PE can be increased, e.g., to 1 mol% (Fig. 6d–f). The fluorescence intensity then passes the detection threshold of the analysis software and all particles are recognized as fluorescent (Fig. 6d–f). However, results from correlated confocal microscopy and AFM (Fig. 8) corroborate that 0.25 mol% NBD-PE is sufficient for effective labelling and tracking of vesicles when using sensitive fluorescence detection systems. Addition of the fluorescent lipid NBD-PE enabled live tracking of PR based proteoliposome systems and may be used for more elaborate types of systems such as nanoreactors henceforth. This will for example open the possibility to follow a population of nanoreactors in complex environmental or biological samples.

Proteoliposomes prepared with the presented reconstitution procedure yield polylamellar vesicles (Fig. 7a) that can be adsorbed onto a flat surface and imaged by AFM without rupturing (Figs 7b and 8). Regular unilamelar liposomes tend to collapse when being adsorbed to mica and are imaged as flat structures by AFM^23^. The observed mechanical stability facilitates immobilization of functionally intact proteoliposomes on surfaces. Biosensing or biotechnological systems are frequently used in conjunction with microfluidic systems relying on immobilized enzymes or nanoreactors^25–27^. The adsorption of proteoliposomes to hydrophilic surfaces is possible by simple physicochemical interactions without the need for surface conjugation or selective affinity components. This could be further improved by adding a targetable feature to the vesicle system that would allow site-specific deposition of nanoreactors.

Based on the chemically switchable proteorhodopsin PR-N221C, we improved the homogeneity and functionality of this essential energizing module for future potential use in more complex biomolecular systems. In this work we laid the foundations for the optical detection and tracking of functional PR-based proteoliposome systems. They can be tracked by NTA in fluid systems or by correlated AFM and confocal microscopy for immobilized samples. We also demonstrated the mechanical stability of polylamellar proteoliposomes that enables adsorption onto flat surfaces and AFM imaging while largely maintaining their structural integrity. This provides some potential applications in processes that rely on the immobilization of catalytic nanoreactors on surfaces including the sensing of biomolecules or the bionanotechnological synthesis of high value chemical compounds^25–27^. The here presented energizing system displays several crucial features that could establish it as a promising starting point for the engineering of more complex nanoreactor systems.

## Supplementary information

Supplementary Information
