# Supplementary material for: Design and assembly of a chemically switchable and fluorescently traceable light-driven proton pump system for bionanotechnological applications
Source: Sci Rep. 2019 Jan 31;9:1046. doi: 10.1038/s41598-018-37260-9 (PMC6355921; doi:10.1038/s41598-018-37260-9)
Supplement: Supplementary file 1 — Supplementary Information [file 41598_2018_37260_MOESM1_ESM.pdf]

Supplementary Information

Design and assembly of a chemically switchable and fluorescently traceable light-driven proton pump system for bionanotechnological applications

S. Hirschi[1], N. Fischer[1], D. Kalbermatter[1], P. R. Laskowski[2], Z. Ucurum[1], D. J. Müller[2], D. Fotiadis[1]

[1]Institute of Biochemistry and Molecular Medicine, University of Bern, Switzerland

[2]Department of Biosystems Science and Engineering, ETH Zürich, Basel, Switzerland

Table S1: Relative reconstitution efficiencies of PR constructs

	$\Delta pH_{Bacteria}$	Expression level (mg/L culture)	Corrected $\Delta pH_{Bacteria}$	$\Delta pH_{Proteoliposomes}$	Relative reconstitution efficiency (%)
**PR-wt**	-0.27	1.5	-0.27	-0.11	100.0
**PRΔ18-C176S-N221C**	-0.19	1.3	-0.22	-0.09	100.4

To estimate the relative reconstitution efficiencies, we compared the proton pumping activities of PR constructs in *E. coli* (corrected for their PR expression levels) with the activities of reconstituted proteoliposomes. To accurately assess the photoactivities of PR constructs, the measured activities in bacteria ($\Delta pH_{Bacteria}$) need to be corrected for the PR expression levels (corrected $\Delta pH_{Bacteria}$). This shows that the PRΔ18-C176S-N221C mutant is slightly less active than the wild-type PR. The ratio of activities for the PR mutant and the wild-type can be compared between bacteria and proteoliposomes, and should be equal, if the relative reconstitution efficiencies are the same (i.e., corrected $\Delta pH_{Bacteria}$(PR-wt) / corrected $\Delta pH_{Bacteria}$(PRΔ18-C176S-N221C) = $\Delta pH_{Proteoliposomes}$(PR-wt) / $\Delta pH_{Proteoliposomes}$(PRΔ18-C176S-N221C). Since the same concentration of PR was used, any deviation can be accounted to different amounts of reconstituted PR. The data suggest that PRΔ18-C176S-N221C was reconstituted with virtually the same efficiency as the wild-type construct.

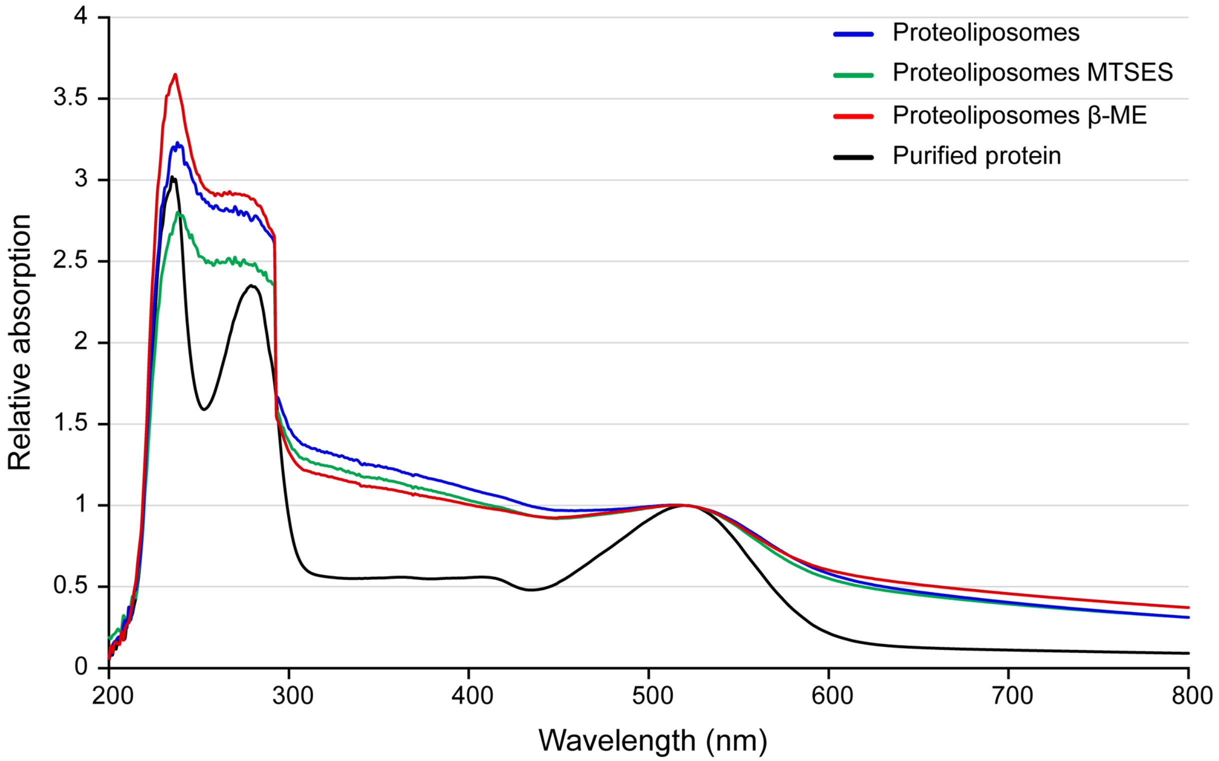

Figure S1: UV-Vis absorption spectra of purified and into proteoliposomes reconstituted PRΔ18-C176S-N221C. Data were normalized to the maximal absorbance of the retinal chromophore peak for better comparison. Maximal absorption wavelengths are 520 nm for purified PRΔ18-C176S-N221C (black), 510 nm for protein reconstituted into liposomes before treatment (blue), 508 nm after MTSES treatment (green) and 509 nm after β-ME treatment (red).

Nanoparticle tracking analysis (NTA) measurements yield size, concentration and refractive intensity of the tracked particles. The refractive intensity indicates a homogeneous distribution in regard to the density of tracked particles (Figure S2), which is equivalent to their lamellarity. Cryo-TEM (Figure 7a) together with the measured refractive intensity from NTA confirm that PR containing proteoliposomes are predominantly polylamellar with a homogeneous unimodal distribution.

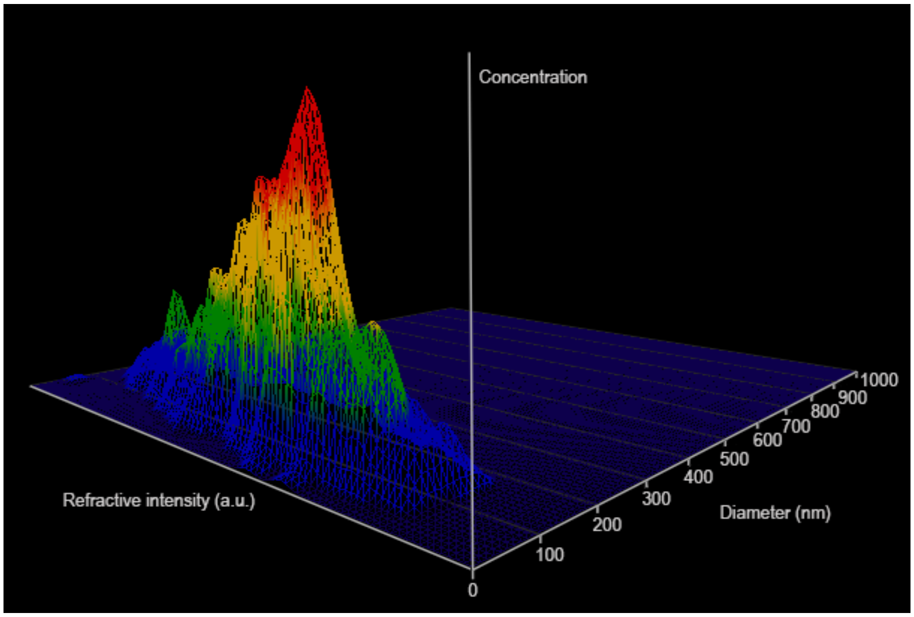

Figure S2: Nanoparticle tracking analysis measurement of PRΔ18-C176S-N221C NBD-PE proteoliposomes. The measurements yield particle size, concentration and refractive intensity. Particles display a homogeneous distribution regarding their refractive intensity and thus their lamellarity.
